# Supplementary material for: Adherence and Effect of Home-Based Rehabilitation with Telemonitoring Support in Patients with Chronic Non-Specific Low Back Pain: A Pilot Study
Source: Int J Environ Res Public Health. 2023 Jan 13;20(2):1504. doi: 10.3390/ijerph20021504 (PMC9860722; doi:10.3390/ijerph20021504)

The comprehensive rehabilitation programme included a combination of several rehabilitation methods recommended in the treatment of LBP, specifically basics of back school, three core stability exercises, two stretching exercises, and a respiratory muscle training exercise.

Back school included basic education about the anatomy of the back, optimal posture, and ergonomics. This information was provided by the physiotherapist.

Core stability exercises – dynamic neuromuscular stabilisation based on neurodevelopmental aspects of motor control (https://www.rehabps.com/)

The first exercise with increasing difficulty level

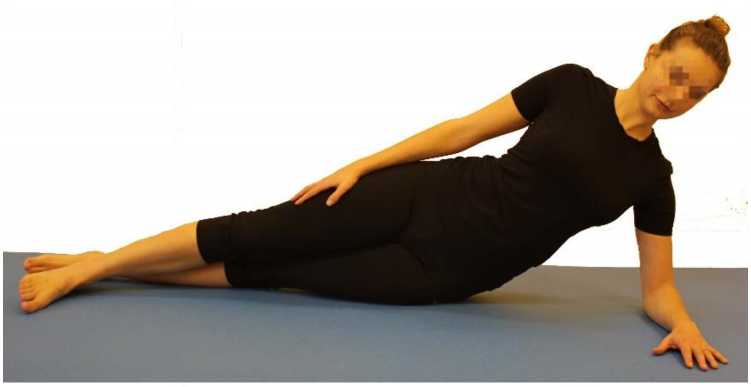

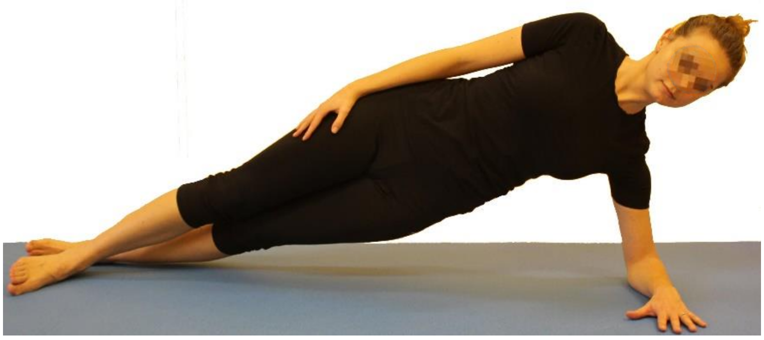

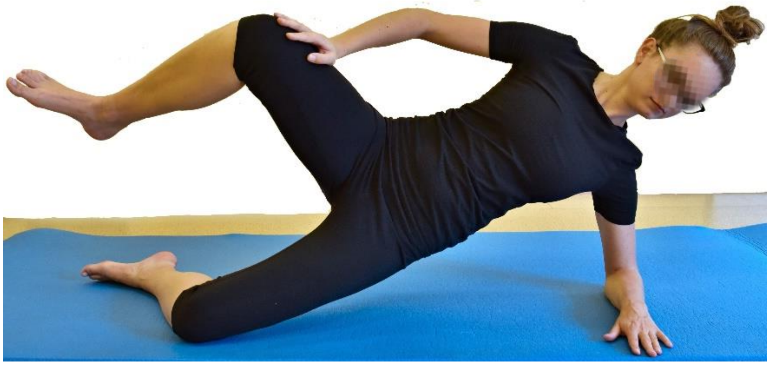

The second exercise with increasing difficulty level

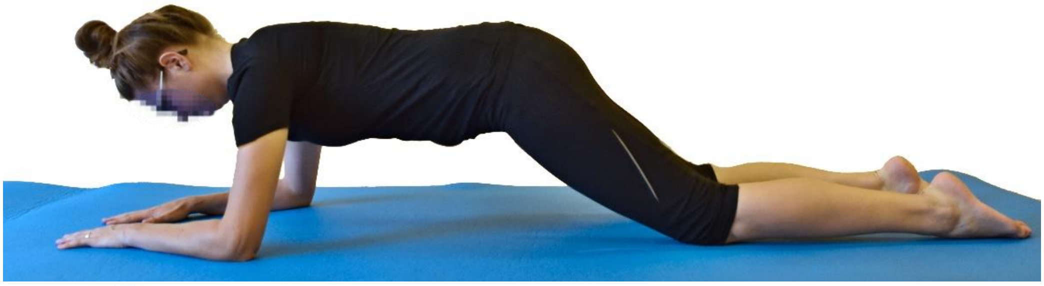

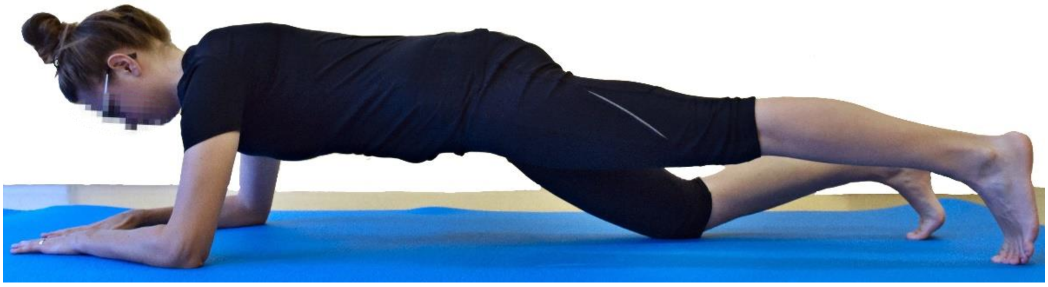

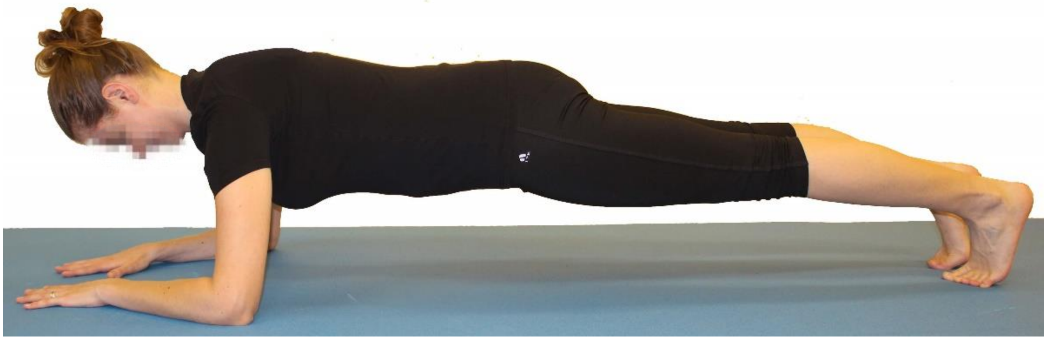

The third exercise

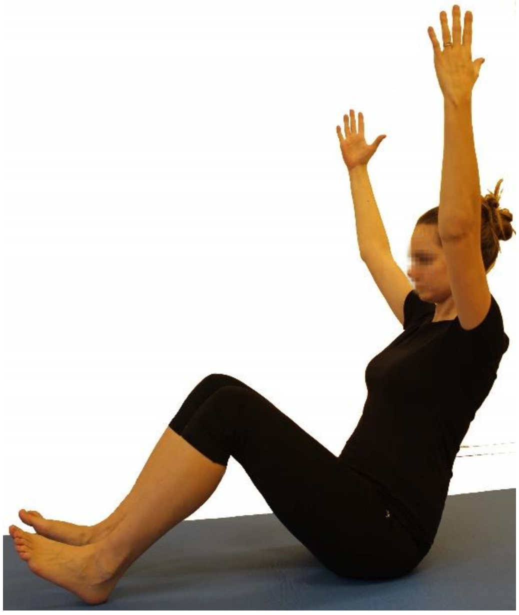

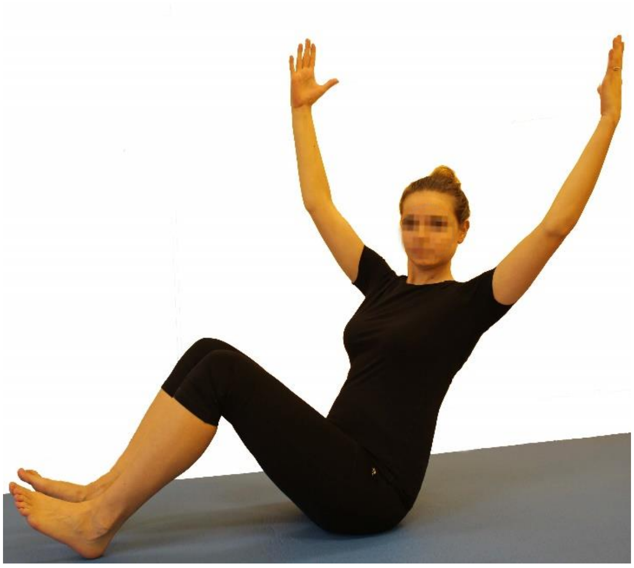

Stretching exercises focused on the low back area

The first stretching exercise

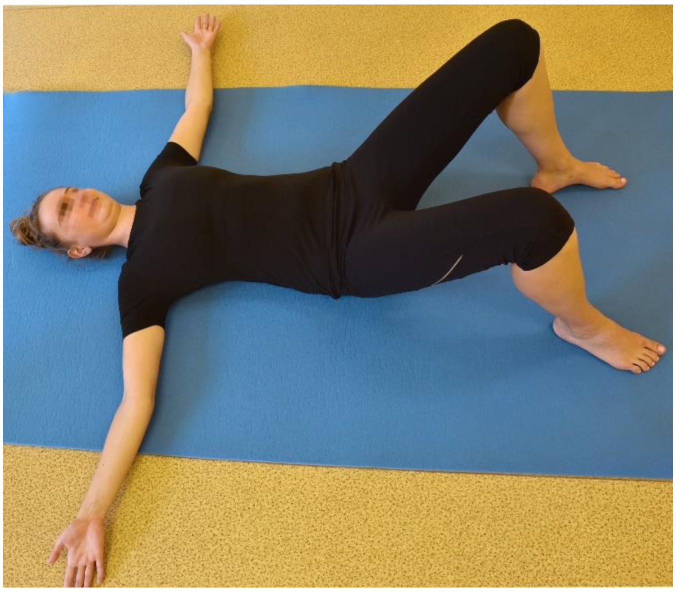

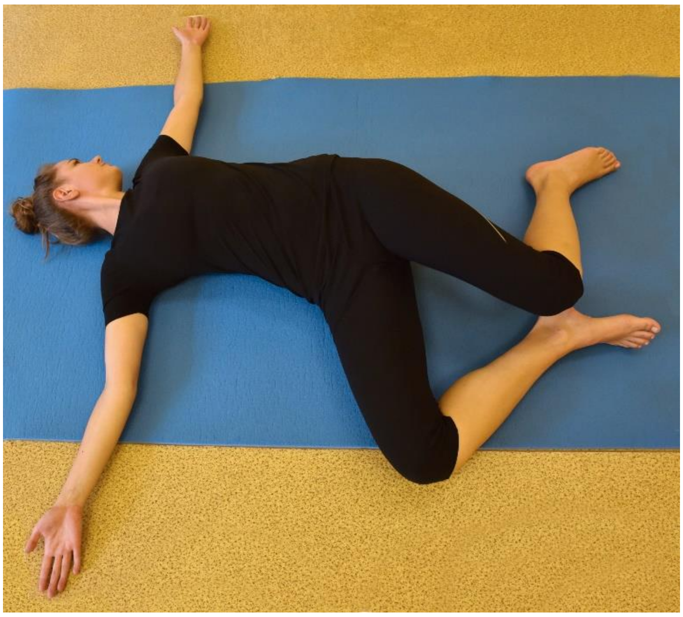

The second stretching exercise

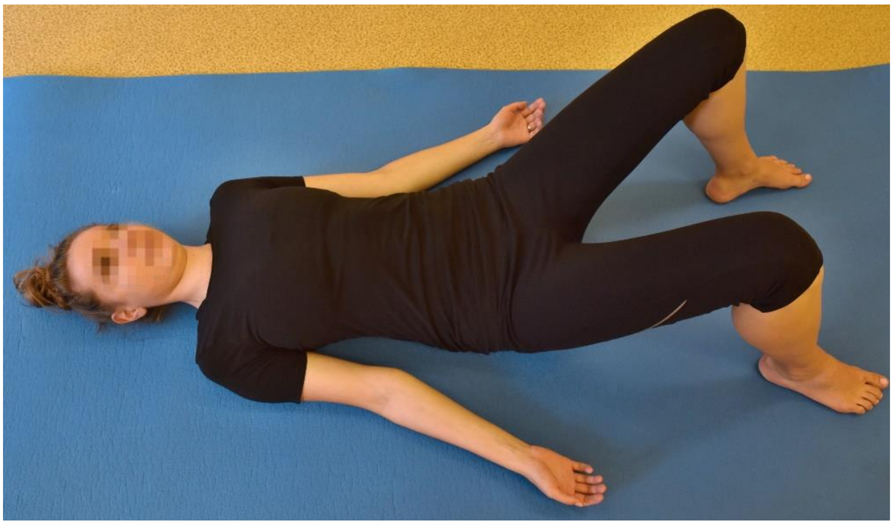

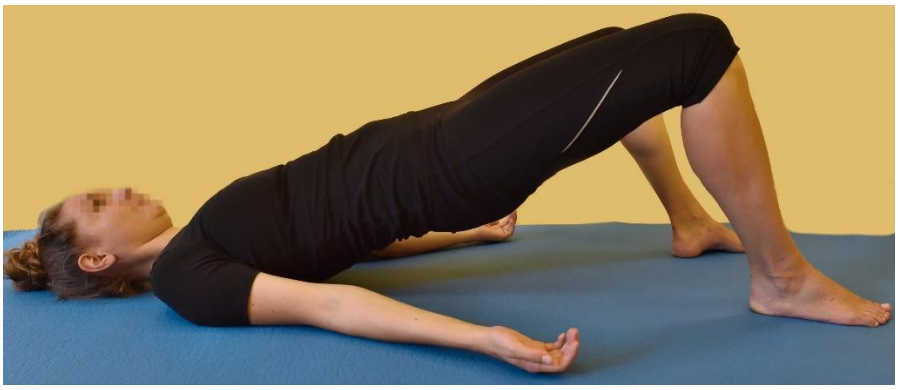

Respiratory muscle training:

Training focused on the correct breathing stereotype

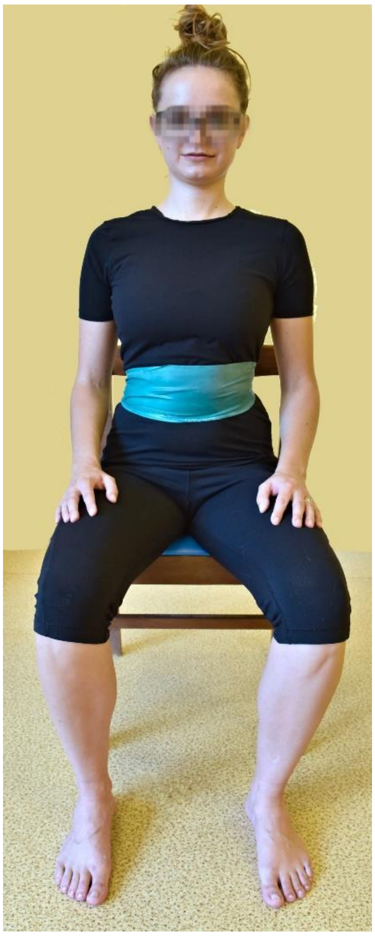

Training with a Threshold IMT and PEP device

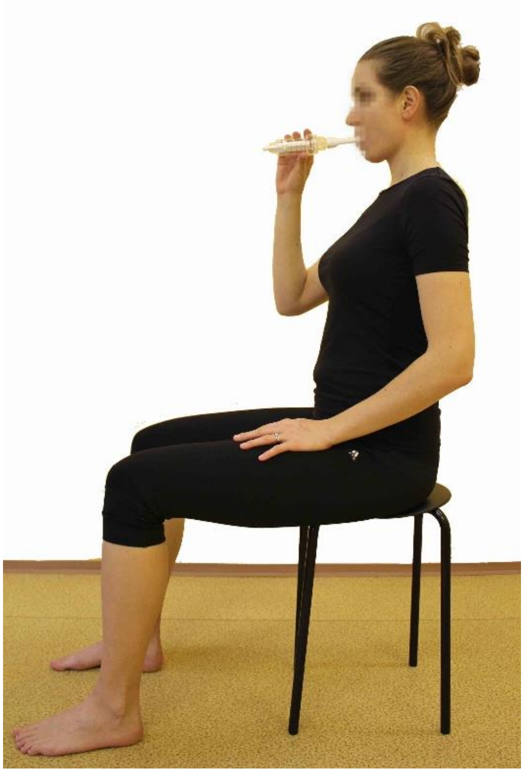

Supplement: Supplementary file 1 [file ijerph-20-01504-s001.zip › ijerph-2076045-supplementary.pdf]
